# Connecting SNPs in Diabetes: A Spatial Analysis of Meta-GWAS Loci

**DOI:** 10.3389/fendo.2015.00102

**Published:** 2015-07-03

**Authors:** William Schierding, Justin M. O’Sullivan

**Affiliations:** ^1^Liggins Institute, University of Auckland, Auckland, New Zealand; ^2^Gravida: National Centre for Growth and Development, University of Auckland, Auckland, New Zealand

**Keywords:** GWAS, epigenetics, DNA folding, gene deserts, meta-analysis, diabetes

## Abstract

Meta-analyses of genome-wide association studies (GWAS) have improved our understanding of the genetic foundations of a number of diseases, including diabetes. However, single nucleotide polymorphisms (SNPs) that are identified by GWAS, especially those that fall outside of gene regions, do not always clearly link to the underlying biology. Despite this, these SNPs have often been validated through re-sequencing efforts as not just tag SNPs, but as causative SNPs, and so must play a role in disease development or progression. In this study, we show how the 3D genome (spatial connections) and *trans*-expression Quantitative Trait Loci connect diabetes loci from different GWAS meta-analyses, informing the backbone of regulatory networks. Our findings include a three-way functional–spatial connection between the TM6SF2, CTRB1–BCAR1, and CELSR2–PSRC1 loci (rs201189528, rs7202844, and rs7202844, respectively) connected through the KCNIP3 and BCAR1/BCAR3 loci, respectively. These spatial hubs serve as an example of how loci in genes with little biological connection to disease come together to contribute to the diabetes phenotype.

## Introduction

The genome-wide association study (GWAS) is a test for statistical associations between common gene variants (single nucleotide polymorphisms, SNPs) and a phenotype. Reductions in overall costs, and sequencing costs specifically, have resulted in a growth in the numbers of GWAS studies and increases in the numbers of samples, phenotype details, and accessibility of the data associated with large consortia studies (1) and re-sequencing validation efforts (2). There is a strong trend for studies to combine data from multiple GWAS studies into a meta-analysis to (1) validate previous findings (2), (2) expand findings from single populations to universal effects (3), and (3) identify novel gene effects (1). Collectively, these changes have increased the power of the GWAS studies, reduced the numbers of false positives, and enabled the detection of small genetic effects that are associated with a number of diseases, including diabetes (1–7).

The large number of previous studies into the genetic contribution to type 2 diabetes (T2D) (2) make it a model phenotype for the application of GWAS meta-analyses to further identify the genetics that underlie the phenotype (2, 3, 5–7). This was recognized by the DIAbetes Genetics Replication And Meta-analysis (DIAGRAM) (1) consortium, which performed a meta-analysis of genetic variants associated with T2D (1). Combined with previous work in diabetes, the DIAGRAM study described 10 new loci (to add to the 56 previously established loci) that are significantly associated with T2D in 34,840 cases in patients of overwhelmingly European descent. However, even with these new loci, only approximately one-third of the established GWAS peaks identified in the DIAGRAM study were validated as significantly associated with a strongly overlapping clinical phenotype; glucose tolerance (*p* < 0.05) (6). The lack of overlap between T2D and glucose tolerance may result from (1) the sample size being insufficient or (2) the GWAS meta-analysis missing aspects of the clinical biology. Given the number of samples and statistical methods used, it is unlikely that the DIAGRAM study was so underpowered that it failed to detect a series of critical SNPs [for review, see Ref. (8)]. Rather, it is more probable that GWAS analyses are lacking information on crucial aspects of the biology of the diabetes. The authors of the DIAGRAM paper even concede that the “difficulties in inferring biological mechanisms from the variants of modest effect identified by GWAS have inhibited progress in defining the pathophysiological basis of disease susceptibility.” (1) Crucially, in its simplest form T2D can be considered as a binary description of metabolic health (i.e., insulin sensitivity). Therefore, the polygenic nature of T2D actually reflects the complexity of the intermingled networks that contribute to metabolism, and the metabolic syndrome. We contend that the GWAS studies are missing the spatio-temporal aspects of the regulatory networks that contribute to the phenotype and remain recalcitrant to detection by standard sequencing and molecular approaches (9).

Single nucleotide polymorphisms identified by GWAS studies may provide critical clues toward unraveling the regulatory network that underlies phenotypic complexity. This is particularly true of SNPs that are highly associated with disease but do not occur in exons or promoters and, as such, have no obvious biological relevance to the disease state (9, 10). It has been estimated that approximately 80% of GWAS SNPs are in regulatory regions (11). These SNPs may represent cell type-specific enhancer sites that bind regulators and contribute to *cis*- or *trans*-gene regulation. For example, obesity-linked non-coding SNPs in the fat mass and obesity-associated gene (FTO) are spatially connected [i.e., they are physically interacting in a manner, which can be captured by proximity-ligation techniques (e.g., 4C-seq)] to promoters at the Iroquois homeobox 3 (IRX3) gene. Crucially, these FTO variants were associated with gene expression changes in IRX3, not FTO (12). Therefore, it has been shown that alterations caused by a SNP (or locus) in region X (not in a gene, but in a regulatory region) can indeed affect the function or regulation of a gene locus in region Y (spatially close, far away in the linear genome sequence, but biologically significant toward a phenotype).

Here, we test the hypothesis that T2D SNPs, which lack obvious functional significance, physically come together (spatially connect) with loci that are significant for the pathophysiology of the disease state. We incorporate a targeted *trans*-expression Quantitative Trait Loci (*trans*-eQTL) analysis to identify those spatial connections that are associated with transcription and, therefore, are putatively regulatory. This analysis identifies distant elements that are significantly associated by gene expression to T2D loci.

## Results

### T2D hubs form by spatially connecting diabetes-related loci

Each gene locus (as defined in methods) can act as a hub for spatial connections within the nucleus. This is exemplified by the spatial organization of the MYRF/FADS1 locus, first associated to T2D in 2010 (5) and re-validated by deep sequencing in 2014 (2). This locus contains rs174535 (chr 11: 61551356), which connects spatially with several loci that are known diabetes risk factors: FADS3 (11q12-q13.1), which regulates desaturation of fatty acids and is clustered with FADS1 and FADS2; RNF214 (11q23.3), which is associated with cardiovascular disease in women with migraines; and USP6 (17p13), which is involved in cell migration and division. The spatial connections of the members of the FADS family are particularly important, given that many variants in this family significantly affect diabetes onset (13). This connection is further supported by the eQTL analysis, as the SNP in the MYRF locus associates with significantly altered expression of the USP6 gene.

### Known T2D SNPs connect in space through intermediary loci

**Figure 1:**
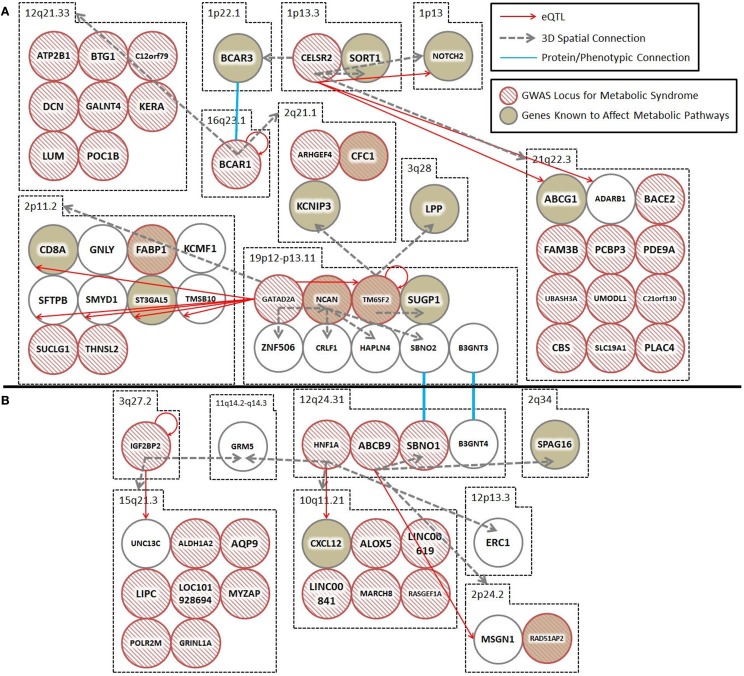
**Spatial and *trans*-expression Quantitative Trait Loci (*trans*-eQTL) connect diabetes loci from different GWAS meta-analyses**. Spatial connections (dashed gray arrows) were obtained from data within the ENCODE database, focusing on those loci that recent meta-analyses have verified as being associated with diabetes. Spatial connections link loci together into a hub(s). These hubs might (1) be regulatory (i.e., contribute to the regulation of nuclear processes including gene regulation, DNA repair, and DNA replication); (2) structural; or (3) simply the result of random associations. To test the hypothesis that some of these spatial connections are regulatory, we included a *trans*-eQTL analysis (red arrows). The *trans*-eQTL analysis highlights significant expression effects associated with the GWAS loci within the spatially associated loci. The eQTL data were derived from a single cancer cell line in the eight Hapmap populations. Future work should determine if these eQTL results are replicated in pancreatic or cardiac cells from individuals developing symptoms of the metabolic syndrome (data currently unavailable). Loci that were identified by GWAS as being important for T2D and the metabolic syndrome are represented by hashed red circles. Loci in biological pathways related to T2D or metabolic syndrome are represented by tan filled loci. Protein/phenotype connections (blue lines) are illustrated where appropriate to connect relevant loci and further expand the regulatory co-interaction diagram. For example, (1) spatial associations with KCNIP3 (2q21) and a physical connection between the BCAR1–BCAR3 proteins link TM6SF2, CTRB1–BCAR1, and CELSR2–PSRC1, into a spatial hub and (2) GRM (11q14) links the IGF2BP2 locus (3q27) to the HNF1A locus (12q24) together along with other loci that have been previously implicated in diabetes. **(A,B)** illustrate two spatial hubs that are connected by functional linkages between SBNO1-SBNO2 and B3GNT3-BGNT4. *For the sake of clarity, the following loci were abbreviated: CELSR2, CELSR2/PSRC1; BCAR1, CTRB1/BCAR1; GATAD2A, GATAD2A/TSSK6/NDUFA13/YJEFN3/CILP2; ABCB9, ABCB9/OGFOD2/PITPNM2.

Spatial (genomic interaction) and functional (protein interaction) connections link three loci (i.e., TM6SF2, CTRB1–BCAR1, and CELSR2–PSRC1, corresponding to rs201189528, rs7202844, and rs599839, respectively) that were identified in two different GWAS meta-studies (Figure 1A). Crucially, these connections are indirectly occurring through intermediary loci (i.e., KCNIP3 – 2q21.1 and BCAR1/BCAR3 – 16q23.1/1p22.1). The TM6SF2 locus is associated with T2D (2), and is involved in the regulation of liver fat metabolism. rs201189528 spatially connects with several other loci: SUGP1 (19p13.11), which affects serum lipid levels and dyslipidemia (13); LPP (3q28), which is associated with T2D in American Indians (13); and KCNIP3 (2q21.1), which is associated with diabetic retinopathy (14). Importantly, KCNIP3 also connects spatially to a T2D SNP (rs7202844, chr 16: 75247391), located within the CTRB1–BCAR1 locus (1).

The third locus, CELSR2–PSRC1 [associated with decreased serum levels of LDL (5)] spatially connects to SORT1 (1p13.3), which is implicated in LDL and triglyceride metabolism (13); NOTCH2 (1p13-p11), which is involved in pancreas development (13) and associated with T2D (1); BCAR3 (1p22.1), which is in the insulin signaling pathway (13); and region 21q22.3, which includes DNMT3L (regulator of methyltransferases, embryonic development, and imprinting), B3GALT5 (implicated in pancreatic cancer) (13), and ABCG1 (cellular lipid homeostasis). The spatial connection to BCAR3 is important here, because this forms the basis for a functional connection between the CTRB1–BCAR1 and CELSR2–PSRC1 loci (SNPs rs7202844 and rs599839). Despite being on different chromosomes, the BCAR1 and BCAR3 gene products tightly interact at their C-terminal domains and the proteins are co-dependent disease progression markers (15).

In summary, rs201189528 (chr 19) is linked to rs599839 (chr 1) through a spatial connection with KCNIP3 (chr 2); meanwhile, rs599839 (chr 1) is spatially connected to BCAR3 (chr 1), whose gene product forms a complex with the BCAR1 gene product (rs7202844). These inter-chromosomal spatial connections and functional protein connection link three highly significant, yet biologically uninformative T2D SNPs with loci previously shown to be components of key diabetes pathways, including LDL and triglyceride metabolism and homeostasis, pancreatic development (and cancer), and insulin signaling.

Spatial connections also connect two T2D-associated loci that were identified in different studies: IGF2BP2 [rs16860235 (1)] and HNF1A [rs1169288 (5)] (Figure 1B). These loci are spatially connected via physical associations with GRM5 (11q14.3), a metabotropic glutamate receptor gene that functions in beta cells and has been associated with both T1D and T2D (13). Compellingly, both rs16860235 and rs1169288 also physically connect to other loci that are involved in energy metabolism and cardiovascular disease (Table 1). Therefore, despite the fact that no direct link has been captured between the IGF2BP2 locus on chromosome 3 and the HNF1A locus on chromosome 12, the shared connection with GRM5 and additional connections to loci that have been previously associated with T2D is consistent with these SNPs associating with a larger hub that links different genes that contribute to T2D.

**Table 1:** **Novel SNPs and their spatial interactions**.

SNP				Spatially linked locus			
SNP (rs)	Position (Chr:bp)	Gene	Reference	Position	Genes	Disease association	Reference
**(i)**
16860235	3:185512361	IGF2BP2	(1)	15q21.3	LIPC and HDLCQ12	Fat metabolism	(13), N
1169288	12:120978847	HNF1A	(5)	10q11.21	PRKG1	Energy metabolism, cellular aging, and late onset diseases (e.g., cardiovascular)	(16, 17), N

**(ii)**
2001844	8:126478745	N/A (40 kb)	(5)	8q24.13	TRIB1	Lipid metabolism and serum lipid levels	(13), N
6909	19:19619542	GATAD2A	(2)	19p12	NCAN	Serum lipid levels and coronary heart disease	N
				2p11.2	IMMT, ST3GAL5, MAT2A, FABP1	Insulin signaling, insulin growth factor and fatty acid metabolism	N
7798124	7:15055616	N/A (>40 kb)	(1)	7p21.1	AGMO (TMEM195)	Decreased glucose-stimulated insulin response, type 2 diabetes	N
				3p12.2		Glycogen storage disease IV	N, G
7168849	15:90346227	ANPEP	(1)	15q26.1	IDDM3	Insulin-dependent diabetes	N
				3q22.1	TF, TOPBP1, NPHP3	Iron homeostasis, pulmonary arterial hypertension, adrenal-hepatic-pancreatic dysplasia.	N, G
7111	15:90373873	AP3S2	(1)	9p31.2	IGFBPL1, IGFBPRP4	Insulin growth factor binding protein genes	N
11755566	6:38116669	ZFAND3	(1)	6q22	FIQTL1	Altered fasting insulin levels	N

**(iii)**
3741530	12:123469647	ABCB9 PITPNM2	(3)	12q24	SBNO1	Coronary artery disease and hypertension	N
				2q34	SPAG16	Childhood obesity in Hispanics	N
				2p24	RAD51AP2	Hypertension in Japanese	N
703977	10:0944230	ZMIZ1	(1)	10q22.3	DUPD1	South Asia populations energy metabolism and weight in females	(18), N
11683087	2:227586606	IRS1	(7)	14q23.3-q24.1	TMEM229B/PLEKHH1	Susceptibility to insulin resistance T2D GWAS	N

*N/A, not applicable, distance to closest gene in brackets. N, NIH GWAS Catalog and NIH Gene Database; G, Genecards and Uniprot*.

The hubs that form about the interacting loci, described above, might be (1) regulatory (i.e., contribute to the regulation of nuclear processes including gene regulation, DNA repair, DNA replication); (2) structural; or (3) simply the result of random associations. To test the hypothesis that some of these spatial connections are regulatory, we included a *trans*-eQTL analysis (Figure 1). The *trans*-eQTL analysis highlights significant expression effects associated with the GWAS loci within the spatially associated loci. Many of the GWAS loci have significant eQTL associations, showing a connection between spatial associations and expression pathways. To highlight one interaction of particular importance, the CELSR2–PSRC1 hub includes several spatial and eQTL associations, including one of each with NOTCH2, which has been previously implicated in diabetes.

### Direct connections form between T2D SNPs

While the spatial connections we have described above have occurred through intermediary loci, direct connections also occur between known SNPs from different studies. For example, the pantothenate kinase 1 (PANK1) locus (2) encodes a gene product for the biosynthesis of coenzyme A that is regulated by p53. PANK1 includes rs10160034 (chr 10: 91352850), which spatially links to 4p16.1. Region 4p16.1 is associated with Wolfram syndrome, an autosomal recessive neurodegenerative disorder characterized by juvenile-onset diabetes mellitus and bilateral optic atrophy (13). rs1801214 (chr 4: 6303022) is associated with T2D (1) and sits within this Wolfram syndrome 1 (WFS1) locus.

### Novel spatial links provide a possible explanation for the T2D association of some SNPs

We have identified clusters of significant SNPs that have putative regulatory and functional relationships to T2D. This spatial clustering may provide a heretofore unrecognized coordination between the different pathways that contribute to T2D. Moreover, we included an analysis of loci that were associated with T2D in non-European populations in an attempt to understand how spatio-temporal analysis can affect ethnic-specific SNPs that underlie a complex disorder like T2D (19) (Table 1).

### Spatial connections link to pancreatic beta cell transcription factors

It remains to be determined whether specific transcription factors or DNA binding proteins are involved in mediating the regulatory spatial interactions that we identified. A preliminary analysis of Pasquali et al. (20) shows that five loci (ANK1, BCAR1, WFS1, ZFAND3, and ZMIZ1) identified in our analysis overlap islet enhancers. Moreover, our spatial analysis included loci with binding sites for four specific beta cell transcription factors [i.e., FOXA2, NKX2.2, NKX6.1, and PDX1 (20)]. CTCF binding sites [a marker implicated in the formation and regulation of spatial interactions in various cell types (21)] were also found in many of our loci.

## Discussion

Genome-wide association study meta-analyses have been growing in popularity, providing a boost in the power to detect SNPs while limiting false positives. However, a GWAS meta-analysis can only analyze genotypes and phenotypes that are homogenous across cohorts, missing any findings that are lost by heterogeneity of methods of detection. This can obscure relationships from GWAS associations to the underlying biology of the phenotype and often there are no obvious features (e.g., genes) that explain the disease risk associated with many SNPs. Here, we have shown that spatial connections better inform how GWAS SNPs outside of T2D genes can interact with other gene regions to associate with T2D risk. In effect, it is possible that the risk associated with non-coding SNPs is attributable to spatio-temporal associations with other genes. The spatial connections that we describe provide clues as to the putative functional connectivity that a series of cross-validated and highly significant SNPs have in T2D.

Single nucleotide polymorphisms that affect the organization of the gene regulatory network within a genome will exhibit temporal and cell-type dependence. In this study, we have identified the existence of specific connections within the human genome. We have not determined the temporal or cell-type specificity of the connections that we identified. More in-depth studies of different cell types [e.g., human lymphoblastoid cells, cervical cancer cells, mammary epithelial cells, umbilical vein endothelial cells, fetal lung fibroblasts, chronic myelogenous leukemia cells, epidermal keratinocytes (21)] will inform on the cellular specificity of the connections that we have identified. While our results provide new avenues for research into the 3D spatio-temporal organization of the genome in diabetes, *in vivo* analyses that incorporate proximity-ligation and empirical perturbation (e.g., using CRISPR–Cas) are required to definitively prove the role for these connections in T2D disease development.

The eQTL analysis also suffers from temporal and cell-type dependence issues. In our current analysis, the presence or lack of eQTL does not definitively (dis)connect two loci. This is due to the cell-line specificity of the eQTL data and the current lack of data for diabetes-specific cell types from individuals at a diabetes-relevant time-point (i.e., pancreatic beta cells from individuals as they are developing insulin resistance). However, as the eQTL analysis is only used to support the spatial analysis, we contend that loci associated by eQTL provide substantial putative evidence of co-regulation and/or spatial connections.

## Experimental Procedures

### Identification of SNPs

We combined results from the six most recent meta-analyses identified from PubMed using the keywords “GWAS,” “meta-analysis,” “diabetes,” “metabolic syndrome,” and “insulin resistance” (Table S1 in Supplementary Material). The six meta-analyses had a total of 124 underlying genetic cohort studies (Table S1 in Supplementary Material).

### Selection of SNPs for analysis – definition of a locus

In total, 489 significant SNPs, identified in the meta-analyses, were used in the downstream analyses (Table S2 in Supplementary Material). SNPs within the same haplotype block were combined and named according to their locus. For our purposes, haplotype blocks were defined as all SNPs sharing linkage disequilibrium coefficients of at least 0.8 *r*^2^ or 0.8 D′. Each haplotype is named according to the genes within the block, thereby known as a locus.

### Literature searches for diabetes connections

Each locus was assessed to determine if it had previously been associated to T2D. Previous associations to diabetes were identified by searches of the published literature (PubMed), disease databases (OMIM), and functional classification databases (Uniprot). In addition, the GWAS Catalog (NHGRI-EBI Catalog of published GWAS) was used to elucidate known GWAS SNPs for each spatial locus, citing any relevant SNPs to the metabolic syndrome that have been found in that locus (Figure 1).

### Identification of spatial connections to diabetes

The web-based programs GWAS3D (22) and HaploRegv2 (23) were used to identify physical connections [as captured by proximity ligation (24, 25)] between T2D SNPs and to identify which SNPs define a locus (see above for criteria).

### Identification of eQTL connections to diabetes

We hypothesized that spatial associations between T2D GWAS SNPs and distant loci were regulatory. Therefore, we set about identifying significant SNP-dependent expression changes within the Hapmap3 dataset (population-adjusted microarray expression values from HapMap lymphoblastoid cell lines in each of the eight Hapmap populations). Significant associations were identified if the expression changes were (1) significant (*p* < 0.05) within at least three of the test populations or (2) significant (*p* < 0.001) within a single population, using a Spearman-rank test through the java-based program Genevar 3.3.0 (26). Thus, the *cis*- and *trans*-eQTLs identify experimentally derived instances where there are significant SNP-dependent expression changes between the two spatially associating loci (i.e., the downstream regulatory effects of the diabetes-associated SNPs).

## Author Contributions

WS, conceived experiments, researched data, and co-wrote the manuscript; JO, conceived experiments and co-wrote the manuscript.

## Conflict of Interest Statement

The authors declare that the research was conducted in the absence of any commercial or financial relationships that could be construed as a potential conflict of interest.

## Supplementary Material

The Supplementary Material for this article can be found online at http://journal.frontiersin.org/article/10.3389/fendo.2015.00102
