# Supplementary material for: Connecting SNPs in Diabetes: A Spatial Analysis of Meta-GWAS Loci
Source: Front Endocrinol (Lausanne). 2015 Jul 3;6:102. doi: 10.3389/fendo.2015.00102 (PMC4490250; doi:10.3389/fendo.2015.00102)
Supplement: Supplementary file 1 [file Data_Sheet_1.PDF]

Supplementary Table 1. *A list of the studies included in the meta-analyses compiled in this study.*

GWAS Meta-Analysis	Study Type	Study Name	Individuals for analysis (Cases/Controls, where available)	Country of origin / Ethnicity	Ref
Teslovich et al.	Community-based cohorts	AGES	2,485	Iceland	(5)
		ARIC	7,841	US	
		MZGWA-AUS	449	Au	
		BLSA	713	US	
		B58C-WTCCC	1,459	UK	
		CHS	3,121	US	
		CoLaus	5,253	Switzerland	
		KORA	1,405	Germany	
		MZGWA-DK	142	Denmark	
		MZGWA-NLD	289	Netherlands	
		EPIC-N-SUBCOH	2,346	UK	
		FENLAND	1,401	UK	
		MZGWA-FIN	137	Finland	
		InCHIANTI	1,134	Italy	
		LOLIPOP	1,599	UK	
		FINRISK	910	Finland	
		NFBC66	5,138	Finland	
		PARC	1,939	US	
		RS-I	5,701	Netherlands	
		RS-II	1,628	Netherlands	
		MZGWA-UK	457	UK	
		SUVIMAX	1,813	France	
		MZGWA-SWE	297	Sweden	

		WGHS	22,041	US	
	Case-control samples	BRIGHT Cases	1,615	UK	
		B58C-T1DGC Controls	2,534	UK	
		DGI Cases	1,528	Finland	
		DGI Controls	1,508	Finland	
		EPIC-N-OBSET Cases	1,078	UK	
		FHS Cases	356	US	
		FHS Controls	394	US	
		FUSION Cases	772	Finland	
		FUSION Controls	982	Finland	
		GENMETS Cases	867	Finland	
		GENMETS Controls	892	Finland	
		MedSTAR Cases	716	US	
		MedSTAR Controls	393	US	
		PennCATH Cases	892	US	
		PennCATH Controls	454	US	
	Family-based samples	ERF	1,108	Netherlands	
		FramHS	7,132	US	
		MICROS	1,037	Austria	
		NSPHS	593	Sweden	
		ORCADES	633	UK	
		SardiNIA	4,184	Italy	
		Vis	771	Croatia	
DIAGRAM Stage 3 (Morris)		Study /stage	n (Cases/Controls)	Ethnicity	(1)
		ARIC /stage1	775/7159	European	
		deCODE /stage1	1465/23194	European (Iceland)	
		DGDG /stage1	679/697	European (France)	

		DGI /stage1	1022/1075	European (Sweden/Finland)	
		EUROSPAN /stage1	269/3710	European	
		FHS /stage1	674/7664	European (USA)	
		FUSION /stage1	1161/1174	European	
		HPFS /stage1	1124/1298	European (USA)	
		KORAGen /stage1	433/1438	European (Germany)	
		NHS /stage1	1467/1754	European (USA)	
		RS1 /stage1	1178/4761	European (Netherlands)	
		WTCCC /stage1	1924/2938	European (UK)	
		AMC-PAS /stage2	48/442	European (Netherlands)	
		BHS /stage2	51/359	European	
		deCODE-Stage2 /stage2	722/10153	European (Iceland)	
		DILGOM /stage2	541/3357	European (Finland)	
		DUNDEE /stage2	3298/3708	European (UK)	
		EAS /stage2	110/641	European	
		EGCUT /stage2	938/915	European (Estonia)	
		EMIL-ULM /stage2	755/1632	European	
		EPIC /stage2	727/927	European (UK)	
		FUSION-Stage2 /stage2	1037/1157	European (Finland)	
		FUSION-D2D2007 /stage2	454/1229	European (Finland)	
		FUSION-DRExtra /stage2	110/785	European (Finland)	
		FUSION-HUNT /stage2	1239/1375	European (Norway)	
		FUSION-METSIM	1169/651	European (Finland)	

		/stage2			
		GMetS /stage2	507/2553	European (France)	
		HNR /stage2	520/3932	European	
		IMPROVE /stage2	898/2521	European	
		KORAGen-Stage2 /stage2	940/4209	European (Germany)	
		PIVUS /stage2	113/864	European (Sweden)	
		PMB /stage2	4976/3500	European (Sweden/Finland)	
		PROMIS /stage2	1178/2472	South Asian (Pakistan)	
		SCARFSHEEP /stage2	341/3073	European (Sweden)	
		STR /stage2	320/1318	European (Sweden)	
		THISEAS /stage2	327/1180	European (Greece)	
		ULSAM /stage2	233/942	European (Sweden)	
		WARREN2 /stage2	1117/4224	European (UK)	
DIAGRAM Ancestry-Specific (Mahajan)		DIAGRAMv3	12171/56862	European	(3)
		AGEN-T2D	6952/11865	East Asian	
		SAT2D	5561/14458	South Asian	
		MAT2D	1804/779	Mexican and Mexican American	
MAGIC (Dupuis)		CHS /stage1	1731	European descent	(6)
		FHS /stage1	6479	European descent	
		TwinsUK /stage1	2224	European descent	
		DGI /stage1	1467	European descent	
		BLSA /stage1	857	European descent	
		FUSION /stage1	1234	European descent	
		SardiNIA /stage1	4305	European descent	

		CoLaus /stage1	5435	European descent	
		GEMS /stage1	1847	European descent	
		InCHIANTI /stage1	1200	European descent	
		deCODE /stage1	6240	European descent	
		NFBC1966 /stage1	4772	European descent	
		NTR / NESDA /stage1	3122	European descent	
		Rotterdam Study /stage1	5974	European descent	
		KORA F4 /stage1	1049	European descent	
		PROCARDIS /stage1	2595	European descent	
		Sorbs /stage1	705	European descent	
		ERF /stage1	918	European descent	
		CROAS (Vis Study) /stage1	720	European descent	
		ORCADES (Orkney) /stage1	719	European descent	
		MICROS (Tyrol) /stage1	1097	European descent	
		ULSAM /stage2	962	European descent	
		PIVUS /stage2	910	European descent	
		ARIC /stage2	7289	European descent	
		Segovia /stage2	1976	European descent	
		Partners/Roche /stage2	1736	European descent	
		Amish (AFDS + HAPI + LS) /stage2	1984	European descent	
		FUSIONs2 /stage2	1000	European descent	
		METSIM /stage2	5983	European descent	
		DIAGEN /stage2	1359	European descent	
		BotniaPPP /stage2	2889	European descent	

		TwinsUK /stage2	1767	European descent	
		Umeå /stage2	2652	European descent	
		SUVIMAX /stage2	1656	European descent	
		DESIR controls /stage2	716	European descent	
		Fenland /stage2	1384	European descent	
		ELY /stage2	1599	European descent	
		Hertfordshire /stage2	2418	European descent	
		WhiteHall II /stage2	4431	European descent	
		BWHHS /stage2	3439	European descent	
		Caerphilly Prospective Study /stage2	960	European descent	
		EFSOCH /stage2	1300	European descent	
		GENDAI /stage2	1047	European descent	
		GHRAS /stage2	627	European descent	
		Health2000 /stage2	5314	European descent	
		MesyBePo /stage2	1130	European descent	
		deCODE /stage2	8060	European descent	
		OBB (Oxford Biobank) /stage2	1163	European descent	
		UKT2DGC /stage2	1,551	European descent	
		FamHS /stage2	550	Caucasian	
		GenomEUtwin /stage2	391	European descent	
		Inter99 /stage2	5778	European descent	
		NHANES /stage2	2309	European descent	
		BHS /stage2	4058	European descent	
		WASHS /stage2	880	European descent	
GUARDIAN (Palmer)	Family-based studies	IRAS-FS /stage 1	3,925	Mexican Americans	(7)
		BetaGene /stage 1		Mexican Americans	

		HTN-IR /stage 1		Mexican Americans	
		MACAD /stage 1		Mexican Americans	
		NIDDM-Athero /stage 1		Mexican Americans	
	Non-family based studies	IRAS /stage 1	411	Mexican Americans	
		TRIPOD /stage 1		Mexican Americans	
	Translation studies	Starr County, TX /stage 2	805/340	Mexican Americans	
		MESA Family /stage 2	81/304	Mexican Americans	
		MESA /stage 2	221/886	Mexican Americans	
		WHI /stage 2	614/2869	Mexican Americans	
		LALES /stage 2	894/467	Mexican Americans	
		SIGMA /stage 2	3848/4366	Mexican Americans	

Supplementary Table 2. *A list of the significant SNPs used in this study to search for spatial connections.*

GWAS Meta-Analysis	SNP	Chr	Position (Build 37 bp)	Risk Allele	Locus	Lead SNP	Trait	Ref
Service et al	rs139816507	1	109656931	A	*KIAA1324*			(2)
	rs667524	1	109656946	C	*KIAA1324*			
	rs1052878	1	109745618	T	*KIAA1324*			
	rs41279704	1	109794123	C	*CELSR2*			
	rs437444	1	109795608	T	*CELSR2*			
	rs200168090	1	109812578	A	*CELSR2*			
	rs77619489	1	109815866	T	*CELSR2*			
	rs7528419	1	109817192	G	*CELSR2*			
	rs12740374	1	109817590	T	*CELSR2*			
	rs660240	1	109817838	T	*CELSR2*			
	rs77579579	1	109818128	T	*CELSR2*			
	rs629301	1	109818306	G	*CELSR2*			
	rs116389032	1	109823457	A	*PSRC1*			
	rs76057315	1	109823537	A	*PSRC1*			
	rs75417653	1	109823574	G	*PSRC1*			
	rs35358959	1	109824250	A	*PSRC1*			
	rs116496512	1	109824503	A	*PSRC1*			
	rs41306199	1	109835163	T	*MYBPHL*			
	rs3850615	1	109839738	A	*MYBPHL*			
	rs76186504	1	109840169	T	*MYBPHL*			
	rs1064599	1	109853935	A	*SORT1*			
	rs461200	1	109855404	G	*SORT1*			

	rs464218	1	109856306	G	*SORT1*		
	rs2228606	1	109878903	G	*SORT1*		
	rs2228604	1	109884775	T	*SORT1*		
	rs72646560	1	109897100	C	*SORT1*		
	rs11142	1	109897103	A	*SORT1*		
	rs181530546	1	109955749	C	*PSMA5*		
	rs62623713	1	110019439	G	*SYPL2*		
	rs114337549	1	110022604	T	*SYPL2*		
	rs12145677	1	110023610	A	*SYPL2*		
	rs34897003	1	230372117	A	*GALNT2*		
	rs3748006	1	230384937	C	*GALNT2*		
	rs2273967	1	230415293	T	*GALNT2*		
	rs15273	1	230415593	T	*GALNT2*		
	rs72647711	1	230415810	A	*GALNT2*		
	rs72647712	1	230415846	C	*GALNT2*		
	rs16851328	1	230416130	A	*GALNT2*		
	rs13728	1	230416218	C	*GALNT2*		
	rs72647716	1	230416729	A	*GALNT2*		
	rs1043900	1	230416744	A	*GALNT2*		
	rs16851339	1	230416832	A	*GALNT2*		
	rs11800118	1	230416950	T	*GALNT2*		
	rs72647719	1	230417014	A	*GALNT2*		
	rs1043908	1	230417294	G	*GALNT2*		
	rs72647722	1	230417321	A	*GALNT2*		
	rs1043941	1	230417557	T	*GALNT2*		
	rs1043944	1	230417560	A	*GALNT2*		
	rs1260326	2	27730940	T	*GCKR*		

	rs183739892	2	27746184	T	*GCKR*		
	rs35422033	2	43571347	A	*THADA*		
	rs111983293	2	43802153	C	*THADA*		
	rs12623703	2	43927459	G	*PLEKHH2*		
	rs10165660	2	43993448	C	*PLEKHH2*		
	rs141721223	2	43993509	T	*PLEKHH2*		
	rs61187167	2	43993722	T	*PLEKHH2*		
	rs13522	2	43994697	A	*PLEKHH2*		
	rs2288709	2	44004010	C	*DYNC2LI1*		
	rs9309107	2	44021826	A	*DYNC2LI1*		
	rs11556157	2	44028013	T	*DYNC2LI1*		
	rs8302	2	44036913	C	*DYNC2LI1*		
	rs10186552	2	44036922	C	*DYNC2LI1*		
	rs77105521	2	44039733	T	*ABCG5*		
	rs2278356	2	44039875	C	*ABCG5*		
	rs140899003	2	44047136	C	*ABCG5*		
	rs6756629	2	44065090	A	*ABCG5*		
	rs11887534	2	44066247	C	*ABCG8*		
	rs56132765	2	44078853	A	*ABCG8*		
	rs9282575	2	44079606	A	*ABCG8*		
	rs137852987	2	44099233	A	*ABCG8*		
	rs145756111	2	44102302	A	*ABCG8*		
	rs4494798	2	44161960	C	*LRPPRC*		
	rs201360053	2	44190687	A	*LRPPRC*		
	rs4953042	2	44201376	C	*LRPPRC*		
	rs4496303	2	169312974	A	*LASS6*		
	rs192072777	2	169627103	G	*LASS6*		

	rs16855819	2	169627271	G	*LASS6*
	rs13001593	2	169629155	A	*LASS6*
	rs114162572	2	169630120	G	*LASS6*
	rs17198917	2	169630304	A	*LASS6*
	rs3732031	2	169681151	T	*NOSTRIN*
	rs34073293	2	169716114	C	*NOSTRIN*
	rs62176781	2	169727863	G	*SPC25*
	rs138726309	2	169763262	T	*G6PC2*
	rs2232323	2	169764141	C	*G6PC2*
	rs496550	2	169779712	T	*ABCB11*
	rs495714	2	169779764	C	*ABCB11*
	rs473351	2	169779896	T	*ABCB11*
	rs497692	2	169789016	T	*ABCB11*
	rs138642043	2	169820801	T	*ABCB11*
	rs2287622	2	169830328	A	*ABCB11*
	rs1121923	8	19809435	A	*LPL*
	rs3289	8	19823192	C	*LPL*
	rs150252331	8	19823988	A	*LPL*
	rs41437944	9	107544673	G	*ABCA1*
	rs2230808	9	107562804	T	*ABCA1*
	rs76881554	9	107578620	A	*ABCA1*
	rs33918808	9	107579632	G	*ABCA1*
	rs2230806	9	107620867	T	*ABCA1*
	rs145183203	9	107646756	A	*ABCA1*
	rs141151519	9	107651444	C	*ABCA1*
	rs3824603	10	91343607	A	*PANK1*
	rs1063943	10	91344169	A	*PANK1*

	rs2292910	11	45903613	A	*CRY2*			
	rs6798	11	45904477	T	*CRY2*			
	rs117665789	11	45924057	T	*MAPK8IP1*			
	rs139448951	11	46774242	T	*CKAP5*			
	rs7928445	11	46784697	C	*CKAP5*			
	rs3829940	11	46879973	A	*LRP4*			
	rs144350829	11	46880732	G	*LRP4*			
	rs146864522	11	46880763	T	*LRP4*			
	rs3816614	11	46890165	C	*LRP4*			
	rs6485702	11	46898771	T	*LRP4*			
	rs10838631	11	46914583	T	*LRP4*			
	rs200129866	11	46920471	C	*LRP4*			
	rs144974139	11	46920492	A	*LRP4*			
	rs3740694	11	47182926	A	*C11orf49*			
	rs79545080	11	47183376	A	*C11orf49*			
	rs78112569	11	47183690	T	*C11orf49*			
	rs3740691	11	47188411	T	*ARFGAP2*			
	rs143049891	11	47259728	A	*DDB2*			
	rs1050244	11	47260477	T	*DDB2*			
	rs4640	11	47261071	T	*ACP2*			
	rs192977216	11	47261441	A	*ACP2*			
	rs145420520	11	47261762	G	*ACP2*			
	rs10838677	11	47267079	T	*ACP2*			
	rs116869426	11	47290967	A				
	rs34602269	11	47298402	G	*MADD*			
	rs3729953	11	47358997	A	*MYBPC3*			
	rs3729989	11	47370041	C	*MYBPC3*			

	rs11570051	11	47371442	A	*MYBPC3*		
	rs3729986	11	47371598	T	*MYBPC3*		
	rs11602837	11	47437033	T	*SLC39A13*		
	rs185398735	11	61567701	G	*FADS1*		
	rs174544	11	61567753	A	*FADS1*		
	rs174545	11	61569306	G	*FADS1*		
	rs174546	11	61569830	T	*FADS1*		
	rs77167250	11	61633947	T	*FADS2*		
	rs1562444	11	92715849	G	*MTNR1B*		
	rs12792653	11	92715916	G	*MTNR1B*		
	rs33984246	11	116660450	G	*APOA5*		
	rs188133936	11	116660653	A	*APOA5*		
	rs34089864	11	116660768	A	*APOA5*		
	rs619054	11	116660813	A	*APOA5*		
	rs185060854	11	116660832	A	*APOA5*		
	rs3135507	11	116661488	T	*APOA5*		
	rs12287066	11	116662331	T	*APOA5*		
	rs3135506	11	116662407	C	*APOA5*		
	rs12721040	11	116691480	A	*APOA4*		
	rs12721043	11	116692293	A	*APOA4*		
	rs2234668	11	116692324	A	*APOA4*		
	rs2241201	12	109992132	G	*MMAB*		
	rs117011771	12	109993148	T	*MMAB*		
	rs111869218	12	109993176	T	*MMAB*		
	rs11067231	12	109993603	A	*MMAB*		
	rs877710	12	109993976	G	*MMAB*		
	rs34368092	12	110019233	A	*MVK*		

	rs201563586	15	58834143	A	*LIPC*			
	rs1529927	16	56904587	C	*SLC12A3*			
	rs118121751	16	56913510	A	*SLC12A3*			
	rs12708965	16	56936319	T	*SLC12A3*			
	rs147630132	16	56974077	C	*HERPUD1*			
	rs34531240	16	57060340	C	*NLRC5*			
	rs28438857	16	57060353	C	*NLRC5*			
	rs13339199	16	57060724	A	*NLRC5*			
	rs117555414	16	57113168	A	*NLRC5*			
	rs201836836	16	57115068	A	*NLRC5*			
	rs27194	16	57116514	T	*NLRC5*			
	rs3729770	19	19257680	T	*MEF2B*			
	rs62135502	19	19303185	A	*RFXANK*			
	rs72997200	19	19307797	T	*RFXANK*			
	rs2228603	19	19329924	T	*NCAN*			
	rs72999033	19	19366632	T	*HAPLN4*			
	rs55765017	19	19368264	A	*HAPLN4*			
	rs75590738	19	19368482	A	*HAPLN4*			
	rs201639037	19	19377344	A	*TM6SF2*			
	rs58542926	19	19379549	T	*TM6SF2*			
	rs201189528	19	19380563	C	*TM6SF2*			
	rs11555053	19	19390185	A	*SF4*			
	rs2285626	19	19467545	T	*KIAA0892*			
	rs2285628	19	19467996	A	*KIAA0892*			
	rs13964	19	19468710	C	*KIAA0892*			
	rs15622	19	19468734	G	*KIAA0892*			
	rs141914615	19	19469200	T	*KIAA0892*			

	rs1063966	19	19616742	A	*GATAD2A*			
	rs1054284	19	19616953	G	*GATAD2A*			
	rs34667451	19	19617017	T	*GATAD2A*			
	rs2033481	19	19617134	A	*GATAD2A*			
	rs41313155	19	19617655	C	*GATAD2A*			
	rs1054308	19	19617656	A	*GATAD2A*			
	rs144529902	19	19618277	T	*GATAD2A*			
	rs10282	19	19619317	C	*GATAD2A*			
	rs6909	19	19619542	G	*GATAD2A*			
	rs1036215	19	19657198	T	*CILP2*			
	rs144960029	19	19672596	T	*PBX4*			
	rs113788698	19	19681089	A	*PBX4*			
	rs145986311	19	19737387	A	*LPAR2*			
	rs140427399	19	19747748	T	*GMIP*			
	rs74497425	19	19757073	C	*ATP13A1*			
	rs187233142	19	19779656	T				
	rs4808209	19	19790159	C	*ZNF101*			
	rs33967144	19	19790255	T	*ZNF101*			
	rs144333705	19	19790421	C	*ZNF101*			
	rs35358946	19	19790690	T	*ZNF101*			
	rs12973901	19	19823270	A	*ZNF14*			
Teslovich Et Al 2010	rs12027135	1	25775733		TMEM57	rs12027135	TC	(5)
	rs12027135	1	25775733		TMEM57	rs12027135	LDL	
	rs4660293	1	40028180		PABPC4	rs4660293	HDL	
	rs2479409	1	55504650		570bp 5' of PCSK9	rs2479409	TC	
	rs2479409	1	55504650		570bp 5' of PCSK9	rs2479409	LDL	

rs3850634	1	63050598		DOCK7	rs2131925	TC	
rs3850634	1	63050598		DOCK7	rs2131925	LDL	
rs2131925	1	63025942		DOCK7	rs2131925	TG	
rs7515577	1	93009438		EVI5	rs7515577	TC	
rs629301	1	109818306		CELSR2	rs629301	TC	
rs629301	1	109818306		CELSR2	rs629301	LDL	
rs1689800	1	182168885		GS1-122H1.2	rs1689800	HDL	
rs2807834	1	220970593		MARC1	rs2642442	TC	
rs2807834	1	220970593		MARC1	rs2642442	LDL	
rs4846914	1	230295691		GALNT2	rs4846914	HDL	
rs1321257	1	230305312		GALNT2	rs4846914	TG	
rs514230	1	234858597		2.6kb 5' of RP4-781K5.8	rs514230	TC	
rs514230	1	234858597		2.6kb 5' of RP4-781K5.8	rs514230	LDL	
rs1367117	2	21263900		APOB	rs1367117	TC	
rs1367117	2	21263900		APOB	rs1367117	LDL	
rs1042034	2	21225281		APOB	rs1367117	HDL	
rs1042034	2	21225281		APOB	rs1367117	TG	
rs1260326	2	27730940		GCKR	rs1260326	TC	
rs1260326	2	27730940		GCKR	rs1260326	TG	
rs4299376	2	44072576		ABCG8	rs4299376	TC	
rs4299376	2	44072576		ABCG8	rs4299376	LDL	
rs6759321	2	136322676		R3HDM1	rs7570971	TC	
rs12328675	2	165540800		COBLL1	rs12328675	HDL	
rs10195252	2	165513091		COBLL1	rs10195252	TG	
rs1515100	2	227128917		79kb 5' of AC068138.1	rs2972146	HDL	
rs2943645	2	227099180		49kb 5' of AC068138.1	rs2972146	TG	

	rs2290159	3	12628920		RAF1	rs2290159	TC	
	rs645040	3	135926622		11kb 5' of MSL2	rs645040	TG	
	rs442177	4	88030261		AFF1	rs442177	TG	
	rs13107325	4	103188709		SLC39A8	rs13107325	HDL	
	rs6450176	5	53298025		ARL15	rs6450176	HDL	
	rs9686661	5	55861786		AC022431.2	rs9686661	TG	
	rs12916	5	74656539		HMGCR	rs12916	TC	
	rs12916	5	74656539		HMGCR	rs12916	LDL	
	rs6882076	5	156390297		30bp 5' of TIMD4	rs6882076	TC	
	rs6882076	5	156390297		30bp 5' of TIMD4	rs6882076	LDL	
	rs1553318	5	156479323		HAVCR1	rs6882076	TG	
	rs3757354	6	16127407		1.9kb 5' of MYLIP	rs3757354	TC	
	rs3757354	6	16127407		1.9kb 5' of MYLIP	rs3757354	LDL	
	rs1800562	6	26093141		HFE	rs1800562	TC	
	rs1800562	6	26093141		HFE	rs1800562	LDL	
	rs3177928	6	32412435		HLA-DRA	rs3177928	TC	
	rs3177928	6	32412435		HLA-DRA	rs3177928	LDL	
	rs2247056	6	31265490		XXbac-BPG248L24.13	rs2247056	TG	
	rs2814982	6	34546560		8.5kb 3' of C6orf106	rs2814982	TC	
	rs2814944	6	34552797		2.3kb 3' of C6orf106	rs2814944	HDL	
	rs9488822	6	116312893		FRK	rs9488822	TC	
	rs11153594	6	116354591		FRK	rs9488822	LDL	
	rs605066	6	139829666		35kb 5' of RP11-12A2.3	rs605066	HDL	
	rs1564348	6	160578860		SLC22A1	rs1564348	TC	
	rs1564348	6	160578860		SLC22A1	rs1564348	LDL	
	rs1084651	6	161089817		2.4kb 5' of LPA	rs1084651	HDL	
	rs2285942	7	21582917		DNAH11	rs1267079	TC	

					8	
rs12670798	7	21607352		DNAH11	rs1267079<br>8	LDL
rs2072183	7	44579180		NPC1L1	rs2072183	TC
rs217386	7	44600695		4.3kb 3' of DDX56	rs2072183	LDL
rs13238203	7	72129667		8.4kb 5' of AC091738.2	rs1323820<br>3	TG
rs17145738	7	72982874		1.1kb 3' of TBL2	rs1714573<br>8	HDL
rs7811265	7	72934510		BAZ1B	rs1714573<br>8	TG
rs4731702	7	130433384		14kb 5' of KLF14	rs4731702	HDL
rs2126259	8	9185146		RP11-115J16.1	rs9987289	TC
rs2126259	8	9185146		RP11-115J16.1	rs9987289	LDL
rs9987289	8	9183358		RP11-115J16.1	rs9987289	HDL
rs11776767	8	10683929		PINX1	rs1177676<br>7	TG
rs1961456	8	18255709		NAT2	rs1495741	TC
rs1495743	8	18273300		15kb 3' of NAT2	rs1495741	TG
rs12678919	8	19844222		19kb 3' of LPL	rs1267891<br>9	HDL
rs12678919	8	19844222		19kb 3' of LPL	rs1267891<br>9	TG
rs1030431	8	59311697		12kb 5' of UBXN2B	rs2081687	TC
rs1030431	8	59311697		12kb 5' of UBXN2B	rs2081687	LDL
rs2737229	8	116648565		TRPS1	rs2737229	TC
rs2293889	8	116599199		TRPS1	rs2293889	HDL
rs2954022	8	126482621		RP11-136O12.2	rs2954029	TC
rs2954022	8	126482621		RP11-136O12.2	rs2954029	LDL

	rs10808546	8	126495818		RP11-136O12.2	rs2954029	HDL	
	rs2954029	8	126490972		RP11-136O12.2	rs2954029	TG	
	rs11136341	8	145043543		PLEC	rs11136341	TC	
	rs11136341	8	145043543		PLEC	rs11136341	LDL	
	rs581080	9	15305378		TTC39B	rs581080	TC	
	rs643531	9	15296034		TTC39B	rs581080	HDL	
	rs1883025	9	107664301		ABCA1	rs1883025	TC	
	rs1883025	9	107664301		ABCA1	rs1883025	HDL	
	rs651007	9	136153875		3.3kb 5' of ABO	rs9411489	TC	
	rs649129	9	136154304		3.7kb 5' of ABO	rs9411489	LDL	
	rs10761731	10	65027610		JMJD1C	rs10761731	TG	
	rs2068888	10	94839642		2kb 3' of CYP26A1	rs2068888	TG	
	rs2255141	10	113933886		GPAM	rs2255141	TC	
	rs1129555	10	113910721		GPAM	rs2255141	LDL	
	rs2923084	11	10388782		AMPD3	rs2923084	HDL	
	rs10832963	11	18664241		7.9kb 5' of SPTY2D1	rs10128711	TC	
	rs3136441	11	46743247		F2	rs3136441	HDL	
	rs174550	11	61571478		FADS1	rs174546	TC	
	rs174583	11	61609750		FADS2	rs174546	LDL	
	rs174601	11	61623140		FADS2	rs174546	HDL	
	rs174546	11	61569830		FADS1	rs174546	TG	
	rs964184	11	116648917		ZNF259	rs964184	TC	
	rs964184	11	116648917		ZNF259	rs964184	LDL	
	rs964184	11	116648917		ZNF259	rs964184	HDL	

rs964184	11	116648917		ZNF259	rs964184	TG	
rs7941030	11	122522375		4kb 5' of UBASH3B	rs7941030	TC	
rs7115089	11	122530591		UBASH3B	rs7941030	HDL	
rs11220463	11	126248211		ST3GAL4	rs11220462	TC	
rs11220462	11	126243952		ST3GAL4	rs11220462	LDL	
rs7134375	12	20473758		41kb 3' of RP11-284H19.1	rs7134375	HDL	
rs3741414	12	57844049		INHBC	rs11613352	HDL	
rs11613352	12	57792580		R3HDM2	rs11613352	TG	
rs7134594	12	110000193		MMAB	rs7134594	HDL	
rs11065987	12	112072424		8.4kb 3' of BRAP	rs11065987	TC	
rs11065987	12	112072424		8.4kb 3' of BRAP	rs11065987	LDL	
rs1169288	12	121416650		HNF1A-AS1	rs1169288	TC	
rs1169288	12	121416650		HNF1A-AS1	rs1169288	LDL	
rs4759375	12	123796238		SBNO1	rs4759375	HDL	
rs4765127	12	124460167		ZNF664	rs4765127	HDL	
rs12310367	12	124486678		ZNF664	rs4765127	TG	
rs838880	12	125261593		12bp 3' of SCARB1	rs838880	HDL	
rs2332328	14	24883058		NYNRIN	rs8017377	LDL	
rs2412710	15	42683787		RP11-164J13.1	rs2412710	TG	
rs2929282	15	44245931		FRMD5	rs2929282	TG	
rs1532085	15	58683366		ALDH1A2	rs1532085	TC	
rs1532085	15	58683366		ALDH1A2	rs1532085	HDL	

	rs261342	15	58731153		RP11-355N15.1	rs1532085	TG	
	rs2652834	15	63396867		RP11-69G7.1	rs2652834	HDL	
	rs11649653	16	30918487		3.6kb 3' of CTF1	rs11649653	TG	
	rs3764261	16	56993324		2.1kb 3' of AC012181.1	rs3764261	TC	
	rs247616	16	56989590		1.6kb 5' of AC012181.1	rs3764261	LDL	
	rs3764261	16	56993324		2.1kb 3' of AC012181.1	rs3764261	HDL	
	rs7205804	16	57004889		CETP	rs3764261	TG	
	rs16942887	16	67928042		PSKH1	rs16942887	HDL	
	rs2000999	16	72108093		TXNL4B	rs2000999	TC	
	rs2000999	16	72108093		TXNL4B	rs2000999	LDL	
	rs2925979	16	81534790		CMIP	rs2925979	HDL	
	rs881844	17	37810218		STARD3	rs11869286	HDL	
	rs7206971	17	45425115		C17orf57	rs7206971	TC	
	rs7225700	17	45391804		RP11-290H9.4	rs7206971	LDL	
	rs4148008	17	66875294		ABCA8	rs41488	HDL	
	rs4082919	17	76377482		PGS1	rs4129767	HDL	
	rs7239867	18	47164717		45kb 3' of LIPG	rs7241918	TC	
	rs7241918	18	47160953		42kb 3' of LIPG	rs7241918	HDL	
	rs12967135	18	57849023		18kb 3' of U4	rs12967135	HDL	
	rs7255436	19	8433196		ANGPTL4	rs7255436	HDL	
	rs6511720	19	11202306		LDLR	rs6511720	TC	
	rs6511720	19	11202306		LDLR	rs6511720	LDL	
	rs737337	19	11347493		DOCK6	rs737337	HDL	
	rs10401969	19	19407718		SUGP1	rs10401969	TC	

	rs10401969	19	19407718		SUGP1	rs10401969	LDL	
	rs10401969	19	19407718		SUGP1	rs10401969	TG	
	rs4420638	19	45422946		339bp 3' of APOC1	rs4420638	TC	
	rs4420638	19	45422946		339bp 3' of APOC1	rs4420638	LDL	
	rs4420638	19	45422946		339bp 3' of APOC1	rs4420638	HDL	
	rs439401	19	45414451		1.8kb 3' of APOE	rs439401	TG	
	rs492602	19	49206417		FUT2	rs492602	TC	
	rs386000	19	54792761		6.7kb 3' of MIR4752	rs386000	HDL	
	rs2277862	20	34152782		7.4kb 3' of ERGIC3	rs2277862	TC	
	rs2902940	20	39091487		46kb 3' of SNORD112	rs2902940	TC	
	rs2902941	20	39091514		46kb 3' of SNORD112	rs2902940	LDL	
	rs4297946	20	39811275		RP3-511B24.5	rs6029526	TC	
	rs909802	20	39936815		ZHX3	rs6029526	LDL	
	rs1800961	20	43042364		HNF4A	rs1800961	TC	
	rs1800961	20	43042364		HNF4A	rs1800961	HDL	
	rs6065906	20	44554015		9.3kb 5' of PCIF1	rs6065906	HDL	
	rs4810479	20	44545048		4.3kb 5' of PLTP	rs6065906	TG	
	rs181362	22	21932068		UBE2L3	rs181362	HDL	
	rs5756931	22	38546033		PLA2G6	rs5756931	TG	
DIAGRAM Stage 3 (Morris)	rs10923931	1	120517959	T	NOTCH2			(1)
	rs2075423	1	214154719	G	PROX1			
	rs340874	1	214159256	C	PROX1			
	rs780094	2	27741237	C	GCKR			
	rs10203174	2	43690030	C	THADA			
	rs11899863	2	43618819	C	THADA			
	rs243088	2	60568745	T	BCL11A			

rs243021	2	60584819	A	BCL11A		
rs243019	2	60585806	C	BCL11A		
rs7569522	2	161346447	A	RBMS1		
rs7593730	2	161171454	C	RBMS1		
rs4410242	2	161192070	G	RBMS1		
rs13389219	2	165528876	C	GRB14		
rs3923113	2	165501849	A	GRB14		
rs2943640	2	227093585	C	IRS1		
rs7578326	2	227020653	A	IRS1		
rs1801282	3	12393125	C	PPARG		
rs13081389	3	12289800	A	PPARG		
rs1496653	3	23454790	A	UBE2E2		
rs7612463	3	23336450	C	UBE2E2		
rs12497268	3	64090363	G	PSMD6		
rs831571	3	64048297	C	PSMD6		
rs13059603	3	63827381	A	PSMD6		
rs6795735	3	64705365	C	ADAMTS9		
rs11717195	3	123082398	T	ADCY5		
rs11708067	3	123065778	A	ADCY5		
rs4402960	3	185511687	T	IGF2BP2		
rs1470579	3	185529080	C	IGF2BP2		
rs6769511	3	185530290	C	IGF2BP2		
rs17301514	3	186613409	A	ST64GAL1		
rs16861329	3	186666461	C	ST64GAL1		
rs6819243	4	1293245	T	MAEA		
rs6815464	4	1309901	-	MAEA		
rs4458523	4	6289986	G	WFS1		

rs1801214	4	6303022	T	WFS1		
rs459193	5	55806751	G	ANKRD55		
rs6878122	5	76427311	G	ZBED3		
rs4457053	5	76424949	G	ZBED3		
rs7756992	6	20679709	G	CDKAL1		
rs10440833	6	20688121	A	CDKAL1		
rs9368222	6	20686996	A	CDKAL1		
rs4299828	6	38177667	A	ZFAND3		
rs9470794	6	38106844	T	ZFAND3		
rs3734621	6	39304211	C	KCNK16		
rs1535500	6	39284050	-	KCNK16		
rs17168486	7	14898282	T	DGKB		
rs6960043	7	15052860	C	DGKB		
rs2191349	7	15064309	T	DGKB		
rs849135	7	28196413	G	JAZF1		
rs849134	7	28196222	A	JAZF1		
rs10278336	7	44245363	A	GCK		
rs4607517	7	44235668	A	GCK		
rs17867832	7	126996837	T	GCC1		
rs6467136	7	127164958	A	GCC1		
rs13233731	7	130437689	G	KLF14		
rs972283	7	130466854	G	KLF14		
rs516946	8	41519248	C	ANK1		
rs7845219	8	95937502	T	TP53INP1		
rs896854	8	95960511	T	TP53INP1		
rs3802177	8	118185025	G	SLC30A8		
rs10758593	9	4292083	A	GLIS3		

rs7041847	9	4287466	A	GLIS3		
rs16927668	9	8369533	T	PTPRD		
rs17584499	9	8879118	T	PTPRD		
rs10811661	9	22134094	T	CDKN2A/B		
rs944801	9	22051670	C	CDKN2A/B		
rs10965250	9	22133284	G	CDKN2A/B		
rs17791513	9	81905590	A	TLE4		
rs13292136	9	81952128	C	TLE4		
rs2796441	9	84308948	G	TLE1		
rs11257655	10	12307894	T	CDC123/CAMK1D		
rs12779790	10	12328010	G	CDC123/CAMK1D		
rs12242953	10	70865342	G	VPS26A		
rs1802295	10	70931474	T	VPS26A		
rs12571751	10	80942631	A	ZMIZ1		
rs1111875	10	94462882	C	HHEX/IDE		
rs5015480	10	94465559	C	HHEX/IDE		
rs7903146	10	114758349	T	TCF7L2		
rs2334499	11	1696849	T	DUSP8		
rs163184	11	2847069	G	KCNQ1		
rs231361	11	2691500	A	KCNQ1		
rs231362	11	2691471	G	KCNQ1		
rs5215	11	17408630	C	KCNJ11		
rs1552224	11	72433098	A	ARAP1 (CENTD2)		
rs10830963	11	92708710	G	MTNR1B		
rs1387153	11	92673828	T	MTNR1B		
rs11063069	12	4374373	G	CCND2		
rs10842994	12	27965150	C	KLHDC5		

	rs2261181	12	66212318	T	HMGA2
	rs1531343	12	66174894	C	HMGA2
	rs2612035	12	66192667	G	HMGA2
	rs7955901	12	71433293	C	TSPAN8/LGR5
	rs4760790	12	71634794	A	TSPAN8/LGR5
	rs4760915	12	71634112	T	TSPAN8/LGR5
	rs12427353	12	121426901	G	HNF1A (TCF1)
	rs7957197	12	121460686	T	HNF1A (TCF1)
	rs1359790	13	80717156	G	SPRY2
	rs4502156	15	62383155	T	C2CD4A
	rs7163757	15	62391608	C	C2CD4A
	rs7177055	15	77832762	A	HMG20A
	rs7178572	15	77747190	G	HMG20A
	rs11634397	15	80432222	G	ZFAND6
	rs2007084	15	90345335	G	AP3S2
	rs2028299	15	90374257	C	AP3S2
	rs12899811	15	91544076	G	PRC1
	rs8042680	15	91521337	A	PRC1
	rs9936385	16	53819169	C	FTO
	rs11642841	16	53845487	A	FTO
	rs7202877	16	75247245	T	BCAR1
	rs2447090	17	2298974	A	SRR
	rs391300	17	2216258	T	SRR
	rs11651052	17	36102381	A	HNF1B (TCF2)
	rs4430796	17	36098040	G	HNF1B (TCF2)
	rs11651755	17	36099840	C	HNF1B (TCF2)
	rs12970134	18	57884750	A	MC4R

	rs11873305	18	58049192	A	MC4R		
	rs10401969	19	19407718	C	CILP2		
	rs8182584	19	33909710	T	PEPD		
	rs3786897	19	33893008	A	PEPD		
	rs8108269	19	46158513	G	GIPR		
	rs4812829	20	42989267	A	HNF4A		
DIAGRAM South-Asian (Morris)	rs13389219	2	165528876	C	GRB14		(1)
	rs459193	5	55806751	G	ANKRD55		
	rs516946	8	41519248	C	ANK1		
	rs2796441	9	84308948	G	TLE1		
	rs12571751	10	80942631	A	ZMIZ1		
	rs10842994	12	27965150	C	KLHDC5		
	rs7177055	15	77832762	A	HMG20A		
	rs7202877	16	75247245	T	BCAR1		
	rs12970134	18	57884750	A	MC4R		
	rs10401969	19	19407718	C	CILP2		
DIAGRAM Ancestry-Specific (Mahajan)	rs6813195	4	153520475	C	TMEM154		(3)
	rs9505118	6	7290437	A	SSR1-RREB1		
	rs17106184	1	50909985	G	FAF1		
	rs3130501	6	31136453	G	POU5F1-TCF19		
	rs6808574	3	187740523	C	LPP		
	rs702634	5	53271420	A	ARL15		
	rs4275659	12	123447928	C	MPHOSPH9		
MAGIC (Dupuis)	rs560887	2	169763148	C	G6PC2		(6)
	rs10830963	11	92708710	G	MTNR1B		
	rs4607517	7	44235668	A	GCK		
	rs2191349	7	15064309	T	DGKB-TMEM195		

	rs780094	2	27741237	C	GCKR			
	rs11708067	3	123065778	A	ADCY5			
	rs7944584	11	47336320	A	MADD			
	rs10885122	10	113042093	G	ADRA2A			
	rs174550	11	61571478	T	FADS1			
	rs11605924	11	45873091	A	CRY2			
	rs11920090	3	170717521	T	SLC2A2			
	rs7034200	9	4289050	A	GLIS3			
	rs340874	1	214159256	C	PROX1			
	rs11071657	15	62433962	A	C2CD4B			
	rs11558471	8	118185733	A	SLC30A8			
	rs4506565	10	114756041	T	TCF7L2			
	rs780094	2	27741237	C	GCKR			
	rs35767	12	102875569	G	IGF1			
GUARDIAN (Palmer)	rs10492494	13	74920186	A	KLF12 / LINC00347			(7)
	rs10830963	11	92708710	G	MTNR1B			
	rs10830963	11	92708710	G	MTNR1B			
	rs11683087	2	227586606	G	LOC646736 / IRS1			
	rs1387153	11	92673828	T	FAT3 / MTNR1B			
	rs1387153	11	92673828	T	FAT3 / MTNR1B			
	rs1602084	4	128843480	G	MFSD8			
	rs196701	6	80147187	C	HMGN3 / LCA5			
	rs1978648	2	43371542	T	HAAO / ZFP36L2 / THADA			
	rs2149423	13	36772381	G	CCDC169-SOHLH2 / SOHLH2			
	rs2206734	6	20694884	T	CDKAL1			
	rs2302063	19	3150418	A	GNA15			

	rs3812570	9	139275204	A	SNAPC4			
	rs3847554	11	92668826	A	FAT3 / MTNR1B			
	rs4887140	15	74046663	G	C15orf59 / TBC1D21			
	rs523079	3	187615862	T	BCL6 / LPP			
	rs6719442	2	2722295	A	MYT1L / TSSC1			
	rs6803803	3	180116563	C	PEX5L / TTC14			
	rs780093	2	27742603	T	GCKR			
	rs788338	19	50778543	C	MYH14			
	rs896232	2	2732877	T	MYT1L / TSSC1			
	rs896598	15	74036629	A	C15orf59			
	rs9368222	6	20686996	A	CDKAL1			
